# Comparative Study of Back Surface Fields on the Radiation Tolerance of GaInP Solar Cells Under 1 MeV Electron Irradiation

**DOI:** 10.3390/nano16171071

**Published:** 2026-08-27

**Authors:** Pan Dai, Hao Lan, Kang Yang, Dengshan Cai, Shan Jin, Shulong Lu

**Affiliations:** 1 School of Information Engineering, Huzhou Normal University, Huzhou 313000, China; 2 Suzhou Institute of Nano-Tech and Nano-Bionics, Chinese Academy of Sciences, Suzhou 215123, China

**Keywords:** GaInP solar cell, electron irradiation, AlInP, AlGaInP, III–V multijunction

## Abstract

Back surface field (BSF) materials are key functional layers that suppress rear-surface recombination and regulate carrier transport in III–V solar cells. This work investigates the performance degradation behavior of GaInP solar cells with AlInP and AlGaInP BSF layers under 1 MeV electron irradiation. Through a combination of optoelectronic measurements and TCAD simulations, the carrier transport and recombination mechanisms before and after irradiation are comprehensively analyzed. Although both devices deliver comparable initial photovoltaic performance, distinct degradation trends emerge under high-fluence electron irradiation. After a cumulative fluence of 1 × 10^15^ e/cm^2^, the cell with an AlInP BSF suffers more severe degradation owing to inferior radiation hardness. Irradiation-induced defects reduce the minority-carrier lifetime and enhance Shockley–Read–Hall (SRH) nonradiative recombination, resulting in a 14% drop in short-circuit current density. In contrast, the AlGaInP BSF exhibits favorable radiation tolerance, and the corresponding device undergoes only a 2% loss in short-circuit current density. The influence of the BSF structure on carrier transport and recombination is systematically analyzed, providing experimental and theoretical support for the design of space-grade GaInP top cells.

## 1. Introduction

Long-lifetime and high-reliability on-orbit missions continuously raise requirements for the conversion efficiency, environmental adaptability, and radiation hardness of space photovoltaic power sources. III–V multijunction solar cells, featuring high efficiency and favorable space compatibility, are being developed via various technical routes for next-generation space energy applications [1,2,3]. Nevertheless, planar lattice-matched and inverted metamorphic III–V multijunction cells still dominate practical space power applications [3,4,5,6].

GaInP possesses a tunable bandgap of 1.88–1.95 eV and efficiently absorbs short-wavelength solar photons. As a critical subcell for achieving high open-circuit voltage (V_oc_) and overall system efficiency, it is widely adopted in Ge-based lattice-matched triple-junction, inverted metamorphic (IMM), and semiconductor-bonded (SBT) space multijunction architectures [2,3,7,8]. High-energy electrons and protons in space induce irreversible lattice displacement damage in GaInP materials, producing point defects that form deep-level recombination centers. These defects reduce the minority-carrier lifetime and diffusion length, deteriorating the photovoltaic performance [3,6,9]. Studies show that irradiation-induced SRH nonradiative recombination is one of the main mechanisms responsible for GaInP top-cell performance degradation [10,11,12,13,14]. Relative to GaAs and InGaAs subcells, GaInP exhibits intrinsically superior radiation-hard characteristics. Nevertheless, the rear heterointerface is sensitive to radiation damage; irradiation-induced interfacial defects aggravate interface recombination and become a critical bottleneck limiting the long-term performance retention of GaInP top cells [9,15].

The back surface field (BSF) is a core structure for optimizing GaInP rear-interface properties and regulating carrier transport [16]. Conduction-band offset (CBO) barriers formed by epitaxially grown wide-bandgap heterostructures efficiently hinder minority carrier diffusion to the rear contact and restrain rear-surface recombination, which enhances the collection efficiency of photogenerated carriers. CBO and valence-band offset (VBO) directly govern carrier confinement and transport and are the theoretical basis of BSF band-structure engineering [17].

From a materials perspective, BSF material selection, composition tuning, and heterostructure design play important roles in carrier confinement [18,19,20]. At present, AlInP and AlGaInP are mainstream wide-bandgap candidates for GaInP solar cell BSF layers. The material composition, Al content, and interface quality of the BSF strongly affect both beginning-of-life performance and radiation hardness: an appropriate AlGaInP BSF composition can optimize barrier height, lower interface recombination velocity, mitigate irradiation-induced minority-carrier lifetime loss, and improve performance retention [17,21,22]. Lu et al. optimized the BSF of (Al)GaInP solar cells and improved V_oc_, demonstrating that rational BSF material and band design effectively improve carrier transport [21]. Gao et al. studied the impact of a GaInP BSF on the radiation resistance of GaInP/GaAs/Ge triple-junction cells and showed that an optimized GaInP BSF in the middle subcell reduces irradiation-induced degradation and improves space adaptability [22]. Wang et al. reported that for IMM3J triple-junction solar cells, increasing the aluminum (Al) component of the quaternary AlGaInP top-cell BSF effectively elevates the BSF barrier height, enhances carrier absorption of the GaInP top subcell, and optimizes multi-subcell current matching, thereby mitigating efficiency degradation under 1 MeV electron irradiation at a cumulative fluence of 1 × 10^15^ e/cm^2^ [23]. Li et al. found that an unoptimized (Al)GaInP/AlGaInP BSF interface can exhibit an initial interface recombination velocity as high as 257 cm/s; interface recombination losses are further aggravated after irradiation, forming a major bottleneck for efficiency stability [24].

Although the optimization of GaInP BSF structures has been widely reported, systematic studies on how different BSF designs affect radiation damage mechanisms remain limited. Most prior studies focus on the beginning-of-life photoelectric optimization of single BSF structures or macroscopic device degradation under fixed irradiation conditions, without a systematic comparison of typical BSF material systems. The differentiated correlations among band alignment, interfacial properties, and irradiation-induced defect evolution remain unclear, and the inherent trade-off between static photovoltaic performance and long-term radiation durability has not been fully clarified, leaving a critical research gap for the radiation-hardened design of GaInP top cells.

In this work, using a single-variable epitaxial process, we fabricate two GaInP single-junction solar cells that differ only in BSF material (AlInP versus AlGaInP). Combining current density–voltage (*J–V*) and external quantum efficiency (EQE) measurements with Sentaurus TCAD device simulations, we systematically investigate photoelectric degradation under electron irradiation. By analyzing band-offset characteristics, SRH recombination evolution, and defect formation for the two BSF types, we reveal how BSF interfacial and band parameters jointly regulate radiation hardness, providing experimental support and theoretical guidance for highly reliable, long-lifetime GaInP top cells for space applications.

## 2. Materials and Methods

### 2.1. Device Fabrication

The GaInP single-junction solar cells were grown via molecular beam epitaxy (MBE) at a substrate temperature of 520 °C. The substrate used in this work is a (100)-oriented GaAs substrate with a 0° off-cut and p-type doping with a concentration of ~2 × 10^18^ cm^−3^. The epitaxial layers were grown with a V/III ratio of approximately 30~50, and the growth rate was maintained at 1 μm/h for all the layers. Beryllium (Be) and silicon (Si) were used as the p-type and n-type dopants, respectively. The thickness and doping concentration of the BSF layers (Al_0.52_In_0.48_P and Al_0_._104_Ga_0_._416_In_0_._48_P) were fixed at 30 nm and 2 × 10^18^ cm^−3^, respectively. Starting from the substrate and extending upward, the full epitaxial stack and its constituent layers are shown in Table 1.

After epitaxial growth, the two types of epitaxial wafers were simultaneously fabricated into complete solar cell devices using standard semiconductor processing procedures. A Ti/Pt/Au rear ohmic contact was deposited on the substrate backside by electron-beam evaporation. For front-side device fabrication, standard photolithography and wet-chemical mesa etching were carried out to define electrically isolated cell mesas. A finger-shaped n-type ohmic contact was formed on the n-GaAs cap layer by AuGe/Ni/Au evaporation using a conventional lift-off technique. After removal of the n-GaAs cap layer in the optically active region, an antireflection coating was deposited by RF sputtering. Finally, the processed wafers were diced into individual solar cell chips for optoelectronic characterization and electron irradiation experiments.

### 2.2. Electron Irradiation Experiment

Electron beam irradiation experiments were performed using an ELV-8 electron accelerator (Institute of Nuclear Physics, Novosibirsk, Russia). The facility supports a maximum electron beam energy of 2 MeV with a continuously tunable beam current ranging from 0.01 mA to 10 mA. The flux was adjusted to obtain the desired total fluence within a specific interval of time, and the electron flux was typically in the range of 10^7^–10^12^ electrons/cm^2^/s. In our work, the experiments were conducted at room temperature, and the samples were directly exposed to the electron beam without any shielding foil placed in front of the specimens. An electron energy of 1 MeV was adopted, and the irradiation fluences were set to 5 × 10^13^, 1 × 10^14^, 5 × 10^14^ and 1 × 10^15^ e/cm^2^, with a constant electron flux of 1 × 10^11^ electrons/cm^2^/s. Preliminary characterization revealed that GaInP cells experienced only slight photoelectric degradation at fluences of 1 × 10^14^ and 5 × 10^14^ e/cm^2^. Therefore, this work mainly compares the device characteristics before and after 1 × 10^15^ e/cm^2^ irradiation, which are uniformly denoted as BOL (beginning of life) and post-irradiation conditions in the subsequent analysis.

### 2.3. TCAD Numerical Simulation

The Sentaurus TCAD software (apsys. 2024) was utilized to establish device models and simulate the optoelectronic properties of GaInP solar cells. It provides mature material libraries for III-V phosphide alloys and enables the flexible definition of heterojunction band offsets and irradiation-induced defect recombination parameters for radiation damage simulation. The multilayer epitaxial structure was accurately reconstructed based on the experimentally grown samples. Critical material parameters, including bandgap width, dielectric constant, carrier effective mass and minority-carrier lifetime were adopted from experimental data and universal device simulation standards. Table 2 shows the material parameters adopted in the TCAD simulations. For GaInP, AlGaInP and InAlP, the carrier mobility is calculated as a function of carrier concentration:

(1) $$\mu_{e}=\frac{0.2}{1\;+{\;\left(\frac{n}{5\;\times \;10^{23}}\right)}^{0.9}}$$

(2) $$\mu_{h}=\frac{0.22}{1+{\left(\frac{p}{9.6\;\times \;10^{20}}\right)}^{0.4}}\;$$

For the GaAs material, temperature-dependent mobility expressions are adopted, where T denotes the lattice temperature in kelvins:

(3) $$\mu_{e}=\frac{0.85\;\cdot {\;\left(300/T\right)}^{2.3}}{1\;+{\;\left(\frac{n}{1.69\;\times \;10^{23}}\right)}^{0.436}}$$

(4) $$\mu_{h}=\frac{0.04}{\left[{\left(T/300\right)}^{2.3}+1.6\times 10^{-24}\;\cdot \;p\;\cdot \;{\left(T/300\right)}^{1.5}\right]\;\cdot \;\left[1+{\left(\frac{p}{2.75\;\times \;10^{23}}\right)}^{0.395}\right]}$$

The irradiation-induced lattice damage was characterized by an equivalent minority-carrier lifetime degradation model, which conforms to the physical mechanism that high-energy particle bombardment introduces deep-level defects and aggravates non-radiative SRH recombination inside the device.

The phenomenological lifetime–damage relationship is given by:

1/τ = 1/τ_0_ + Kτ·φ (5)

where τ_0_ is the initial minority-carrier lifetime, Kτ denotes the lifetime damage coefficient, and φ represents the electron irradiation fluence. Kτ can be derived from experimental *J–V* and EQE data following the analytical displacement-damage method reported by Wang et al. [23]. For the AlGaInP-BSF device, $K_{\tau }=2.4\times 10^{-6}\;cm^{2}/s$, and for the AlInP-BSF device, $K_{\tau }=1.35\times 10^{-5}\;cm^{2}/s$. The initial minority-carrier lifetime $\tau_{0}=50\;ns$ of the GaInP absorber layer is adopted from a published irradiation simulation study [25]. The corresponding lifetime values at different irradiation fluences for both device structures are summarized in Table 3. Under electron irradiation, the minority-carrier lifetime can decrease by one or even more orders of magnitude depending on the irradiation fluence. This formulation collectively describes the enhancement of non-radiative SRH recombination induced by irradiation-induced lattice defects.

The primary objective of this numerical simulation is to investigate the relative radiation-induced degradation behavior of two different BSF configurations. To reduce excessive mesh quantities, a heavy computational burden, and convergence difficulties, the simulation adopts a two-dimensional slice structure with a lateral width of 5 μm. The contact regions occupy 2 μm out of the total 5 μm width. In addition, no antireflection coating stack is constructed. Exactly the same geometry and contact conditions are used for both the AlInP-BSF and AlGaInP-BSF models to guarantee a valid comparative analysis.

## 3. Results and Discussion

**Figure 1:**
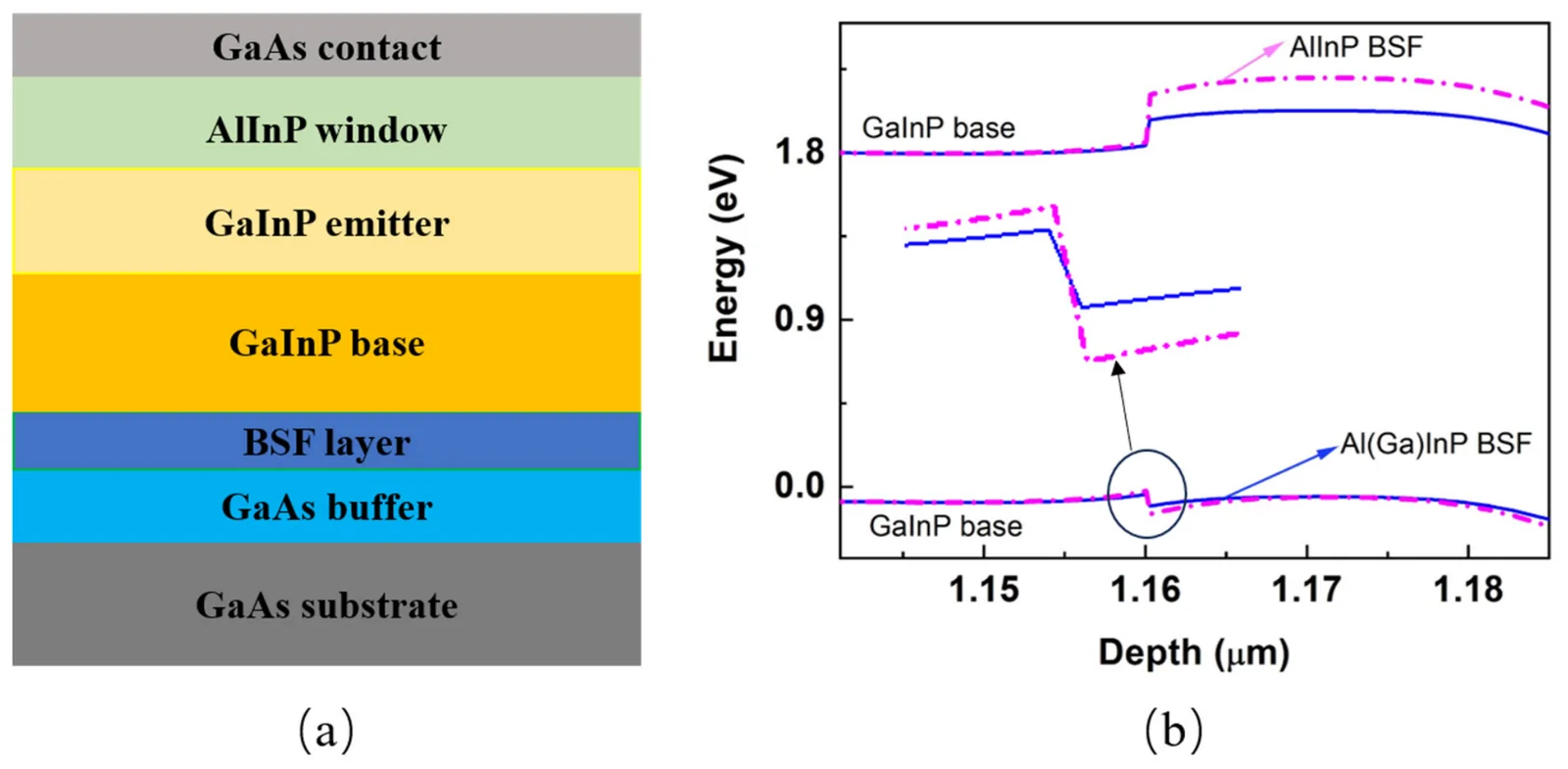
(**a**) Schematic structure of the GaInP solar cell; (**b**) Energy band diagram at the interface between GaInP base and BSF layer.

Figure 1a presents a schematic epitaxial stack diagram of the GaInP solar cell. All functional layer thicknesses and doping specifications follow the fabrication details given in Table 1 and the experimental section. The two GaInP devices adopt identical epitaxial configurations except for the BSF material. Figure 1b shows the energy band diagram at the heterointerface between the GaInP base and the BSF layer. The bandgap and band offsets of the two BSF materials are directly determined by their Al content, which can be quantitatively calculated via heterojunction band offset formulas.

The band offsets were calculated using the electron affinity rule for ideal abrupt heterojunctions, which is widely adopted for III–V heterostructures [26].

(6) $$\Delta E_{C}=\chi_{1}-\chi_{2}$$

(7) $$\Delta E_{V}=\left(E_{g,2}-E_{g,1}\right)-\left(\chi_{1}-\chi_{2}\right)$$

where $\chi_{1}$ and $\chi_{2}$ denote the electron affinities of the two adjacent semiconductor materials; $E_{g,1}$ and $E_{g,2}$ are their respective bandgap energies.

The bandgap of the p^+^-AlGaInP BSF is 2.08 eV, and the bandgap of the GaInP absorber base is 1.88 eV. Both values are calibrated from photoluminescence (PL) measurements on our epitaxial samples. Band-structure calculations yield a conduction band offset ΔE_C_ = 0.06 eV and a valence band offset ΔE_V_ = 0.13 eV for the InGaAlP/GaInP heterojunction. As for the p^+^-AlInP BSF with a bandgap of 2.26 eV paired with the GaInP base layer with a bandgap of 1.88 eV, the heterojunction band offsets are taken as ΔE_C_ = 0.134 eV and ΔE_V_ = 0.25 eV [27,28]. It is clearly observed that both conduction and valence band offsets at the AlInP/GaInP interface are significantly larger. The enhanced energy barriers can more effectively suppress minority-carrier diffusion toward the rear surface.

**Figure 2:**
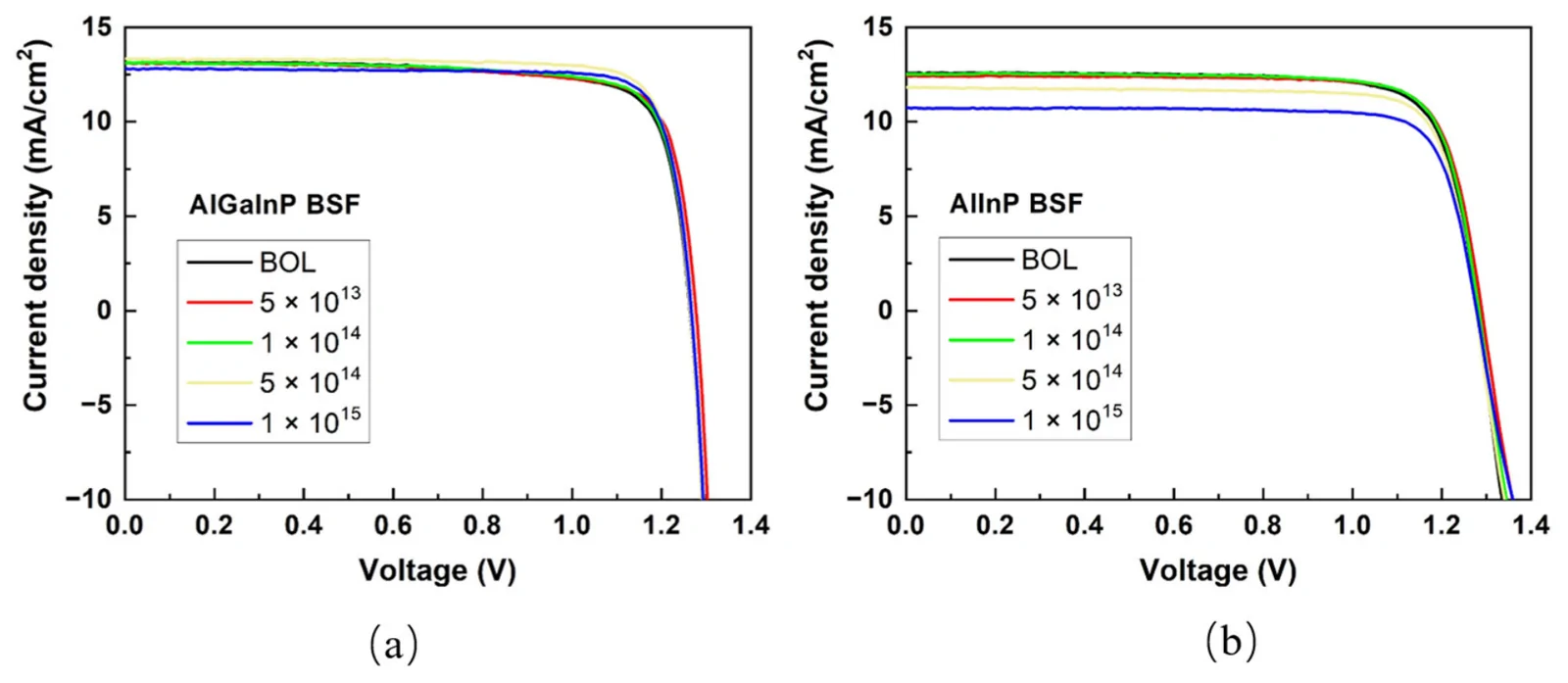
*J–V* characteristics of GaInP solar cells before and after electron irradiation: (**a**) AlGaInP BSF device; (**b**) AlInP BSF device.

Figure 2 shows the *J–V* curves of GaInP solar cells with AlGaP and AlInP BSF structures after graded electron irradiation. The GaInP cell with the AlGaInP BSF delivers higher carrier collection efficiency and superior short-circuit current density (*J_sc_*), yet its valence band barrier is weaker than that of the high-Al-ratio AlInP BSF, leading to a relatively lower V_oc_. This discrepancy originates from the distinct heterojunction band alignments of the two BSF materials [29]. The larger valence band offset of AlInP may create a higher barrier for hole transport and collection, which could contribute to the lower *J_sc_* observed in AlInP-BSF devices.

After electron irradiation, the V_oc_ of both types of cells fluctuates within experimental uncertainty, without statistically significant degradation. The overall performance loss is mainly reflected in *J_sc_*, which is a typical degradation characteristic of III-V top cells under 1 MeV electron irradiation [23]. For the GaInP cell employing AlGaInP BSF, V_oc_ stays at approximately 1.27 V across all investigated irradiation fluences, and the *J_sc_* decreases only slightly. Its initial *J_sc_* before irradiation is 13.12 mA/cm^2^, which drops to 12.79 mA/cm^2^ after 1 MeV 1 × 10^15^ e/cm^2^ electron irradiation, retaining approximately 98% of the original value. For the GaInP solar cell with AlInP BSF, the V_oc_ stays at approximately 1.28 V under all irradiation conditions as well, but its *J_sc_* suffers a severe reduction: the initial value of 12.58 mA/cm^2^ falls to 10.75 mA/cm^2^ after 1 × 10^15^ e/cm^2^ 1 MeV electron irradiation, corresponding to merely 86% of the pre-irradiation level.

At low irradiation fluences, the AlInP BSF possesses a larger heterojunction CBO, which effectively suppresses rear-surface minority-carrier recombination, resulting in milder photocurrent attenuation [30]. Once the irradiation fluence rises to 5 × 10^14^ e/cm^2^ and above, the ternary AlInP lattice tends to generate abundant deep-level point defects under high-energy electron bombardment. Such defect formation drastically weakens its minority-carrier blocking capability [31]. Such differences in defect evolution behavior may stem from differences in atomic displacement threshold energies and defect-related material properties between ternary AlInP and quaternary AlGaInP alloys. In contrast, the Ga constituent in the AlGaInP BSF helps suppress the buildup of irradiation-induced defects, which in turn preserves effective minority-carrier blocking capability at the heterointerface. Consequently, the AlGaInP device exhibits a moderate current-decay trend over the whole fluence range and retains favorable optoelectronic performance under high-dose electron irradiation.

**Figure 3:**
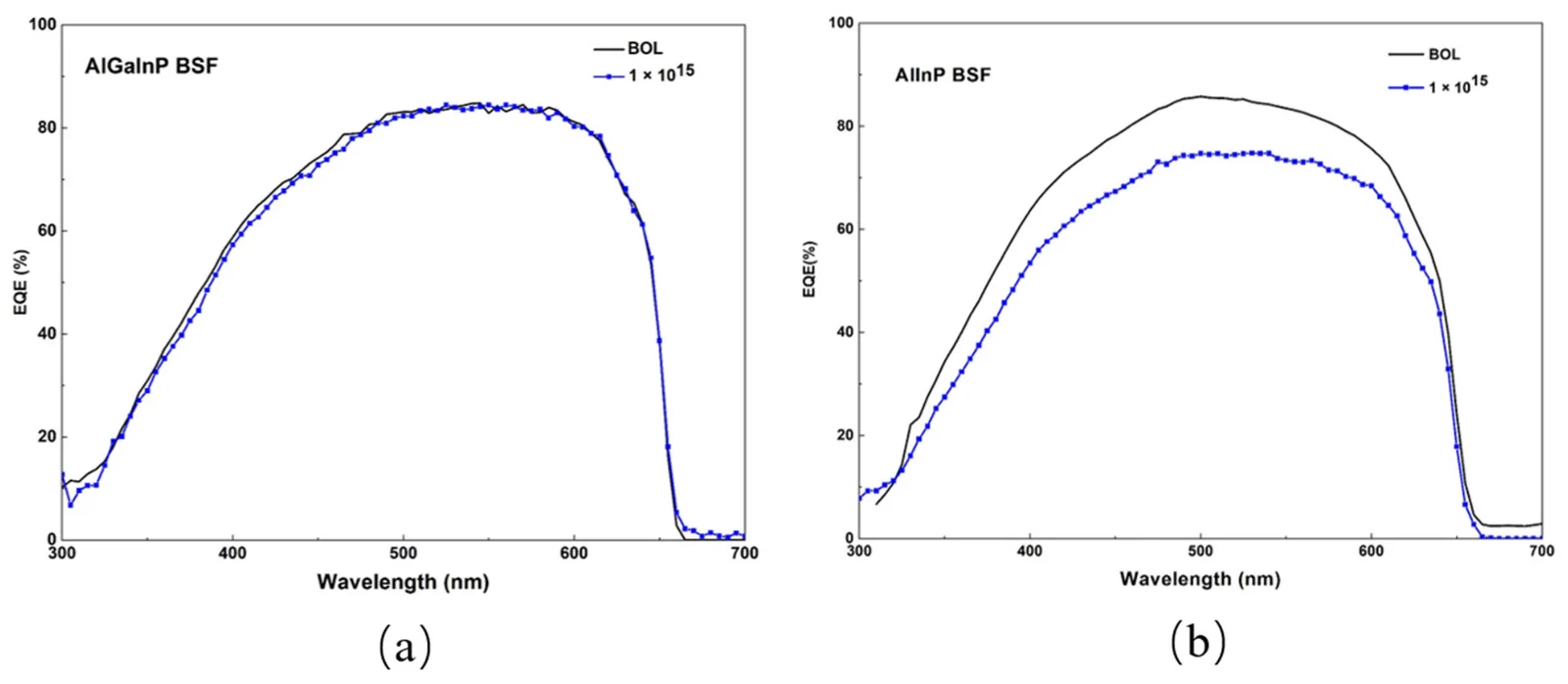
EQE spectra of GaInP solar cells before and after electron irradiation: (**a**) AlGaInP BSF; (**b**) AlInP BSF.

Figure 3 presents the EQE spectra of GaInP solar cells with AlGaInP and AlInP BSFs measured at BOL and after electron irradiation with a fluence of 1 × 10^15^ e/cm^2^. Under the unirradiated condition, the two cells exhibit similar spectral response levels, and the cell with the AlGaInP BSF delivers slightly higher *J_sc_*, which is consistent with the *J–V* measurement results. After electron irradiation of 1 × 10^15^ e/cm^2^, the two types of cells show distinct EQE degradation behavior. For the AlGaInP BSF cell, only slight attenuation of the spectral response is observed across the whole wavelength range, with no obvious loss in the long-wavelength region. The magnitude of photocurrent degradation for our single-junction GaInP cell with an AlGaInP-BSF is at a comparably low level to that of the GaInP top cell reported by Wang et al., despite differences between single-junction and triple-junction device architectures [23].

In contrast, the AlInP BSF cell suffers a dramatic drop in medium- and long-wavelength EQE; the spectral response outside the peak wavelength decreases by more than 12%. The EQE results are consistent with the *J–V* characteristics under irradiation, which collectively demonstrate that the AlInP BSF cell undergoes rapid photocurrent degradation after electron irradiation. This apparent trend seems contrary to the report by Wang et al. [23], where a higher-Al BSF improved radiation tolerance in triple-junction devices. Such a discrepancy mainly arises from device architecture differences: their performance benefit originates from multi-subcell current-matching optimization rather than the intrinsic radiation hardening of the GaInP subcell, which is excluded in our standalone single-junction configuration.

To quantitatively reveal the microscopic mechanisms underlying the distinct irradiation-induced degradation of cells with the two types of BSF layers, a Sentaurus TCAD device simulation model was constructed according to the experimental epitaxial structure. The layer thickness, doping concentrations, and intrinsic material parameters of the ternary and quaternary semiconductors were imported into the model. To describe the damage induced by 1 MeV electron irradiation, a lumped-parameter model based on minority-carrier lifetime was employed to represent irradiation-generated defects. The formation of radiation-induced deep-level recombination centers was mimicked by imposing a fluence-dependent degradation of the minority-carrier lifetime in the corresponding material layers.

The carrier recombination process is dominated by Shockley–Read–Hall (SRH) recombination, and the SRH recombination rate can be expressed as:

(8) $$R_{\mathrm{SRH}}=\frac{np\;-\;n_{i}^{2}}{\tau_{p}\left(n\;+\;n_{1}\right)\;+\;\tau_{n}\left(p\;+\;p_{1}\right)}$$

The minority-carrier lifetime τ decreases monotonically with increasing irradiation fluence, which directly accelerates the non-radiative recombination rate inside the device.

**Figure 4:**
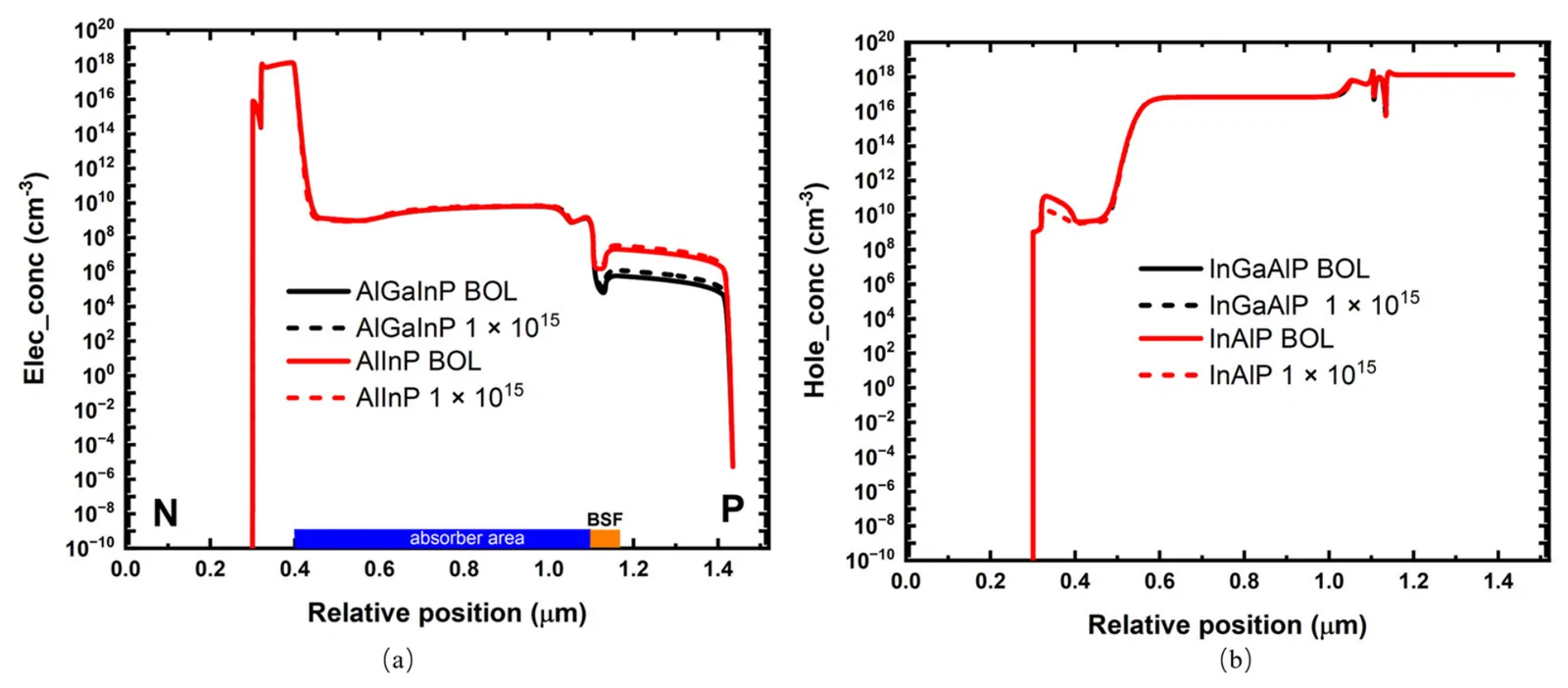
(**a**) Electron concentration and (**b**) hole concentration distributions of GaInP solar cells with different BSF layers before and after electron irradiation.

Figure 4 illustrates the variations in electron and hole concentrations of GaInP solar cells with different BSF layers before and after electron irradiation. Within the GaInP base region at depths smaller than 1.10 μm, the electron concentration curve for the AlInP BSF structure is consistently higher than that for the AlGaInP BSF. This indicates that the AlInP BSF confines photogenerated minority electrons within the base region owing to its larger CBO, which is consistent with the analysis derived from the energy band diagrams.

After irradiation, both cells show enhanced carrier losses. Irradiation introduces defects within the BSF layers, and these defects act as efficient non-radiative recombination centers. As photogenerated electrons migrate toward the rear heterointerface, recombination-induced carrier losses become aggravated. Throughout irradiation, the AlInP BSF device inherently maintains higher electron concentration in the BSF region—its carrier density is two orders of magnitude higher than that of its AlGaInP counterpart. The abundant electrons accumulated in the AlInP BSF are more prone to SRH recombination through radiation-induced defects, resulting in severe localized carrier losses. Such enhanced non-radiative recombination reshapes the spatial distribution of photogenerated carriers and largely accounts for the overall device performance degradation. For a given BSF structure, irradiation-induced charged defects modify the local space-charge distribution and the heterointerface-related electric field, bringing more photogenerated electrons into the BSF region and further enhancing SRH recombination together with a higher trap density. In conclusion, the enhanced recombination originates from the combined effect of an elevated electron concentration and the irradiation-induced reduction in minority-carrier lifetime.

Benefiting from its larger conduction-band offset, the AlInP BSF is capable of confining minority electrons within the GaInP base region under pristine conditions. Nevertheless, this intrinsic feature loses its beneficial effect under irradiation. Compared with the AlGaInP counterpart, the AlInP BSF retains a two-orders-of-magnitude higher electron concentration throughout irradiation. Such abundant accumulated electrons are highly susceptible to SRH recombination via radiation-induced defects, causing severe localized carrier losses. This observation may be linked to the intrinsic material properties of AlInP and AlGaInP themselves.

It should be noted that our device-scale experiments and drift-diffusion TCAD simulations cannot provide direct atomistic-scale proof of defect evolution. Atomistic-level interpretations from the published literature can provide clues supporting our qualitative inferences [32,33,34,35]. The inferior radiation hardness of the AlInP BSF can be qualitatively attributed to differences in displacement threshold energy, defect introduction rate, and defect annealing behavior. In the AlInGaP material system, the displacement threshold energy ($E_{d}$) of phosphorus atoms is markedly lower than the conventional reference value of 8 eV established for InP [33]. As the Al composition increases toward AlInP, the phosphorus $E_{d}$ decreases further [34], facilitating more efficient generation of phosphorus vacancy–phosphorus interstitial ($V_{P}-P_{i}$) Frenkel pairs under 1 MeV electron irradiation. In terms of defect introduction behavior, the total introduction rate of majority-carrier trap centers for AlGaInP is reported to be 0.39 cm^−1^, while the carrier-removal rate equals 1 cm^−1^ [35]. For AlInP, its lower phosphorus $E_{d}$ is expected to result in significantly higher defect-introduction and carrier-removal rates, which is consistent with the observed severe Jsc degradation. In addition, the phosphorus vacancy-related deep level in AlInP (activation energy = 0.65 eV [36]) and the H2 defect in AlInGaP (annealing activation energy = 0.60 eV, associated with $V_{P}-P_{i}$ Frenkel pairs [35]) share the same microscopic origin. However, the AlInP lattice likely exhibits higher migration barriers for vacancies and interstitials, suppressing Frenkel-pair recombination and yielding a higher net concentration of electrically active radiation-induced defects.

**Figure 5:**
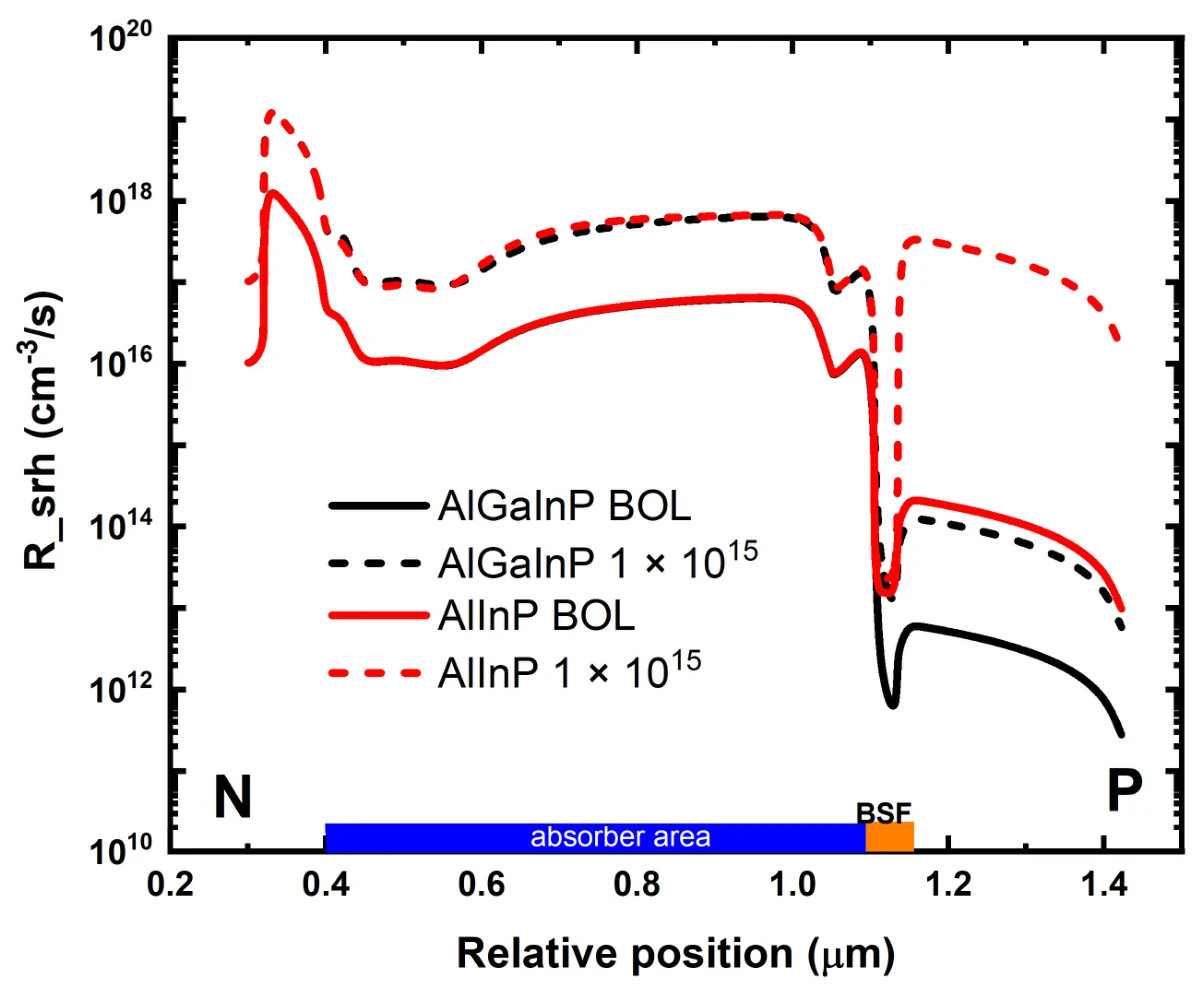
SRH recombination rate distribution of GaInP solar cells with different BSF layers before and after electron irradiation.

A small fraction of photogenerated electrons can transport into the back surface field layer. Upon irradiation, these electrons are rapidly captured by irradiation-induced deep-level defects and trigger SRH non-radiative recombination. This loss cannot be directly identified from electron concentration distribution profiles and requires a joint quantitative analysis with the SRH recombination rate distribution (Figure 5). The baseline SRH recombination rate of the AlGaInP BSF is extremely low before irradiation. Even though its recombination rate increases significantly after irradiation, the absolute value over the whole region remains far lower than the intrinsic recombination level of the AlInP BSF before irradiation. In contrast, the AlInP BSF already exhibits a relatively high recombination rate in the pristine state. After electron irradiation introduces abundant deep-level defects within the bandgap, the electron and hole lifetimes are further shortened, and the SRH recombination rate maintains an extremely high magnitude. Consequently, it suffers a considerably larger overall non-radiative carrier loss compared with the AlGaInP BSF. At the heterointerface between the back surface field and the rear contact layer, the peak recombination rate of the irradiated AlInP BSF is one order of magnitude higher than that of the AlGaInP BSF. This indicates that heavy non-radiative loss occurs at this rear heterointerface. A large number of photogenerated electrons arriving at this region are captured and undergo SRH recombination, which drastically reduces the carrier collection efficiency and leads to a substantial drop in *J_sc_* for GaInP solar cells with an AlInP BSF under high-dose irradiation.

**Figure 6:**
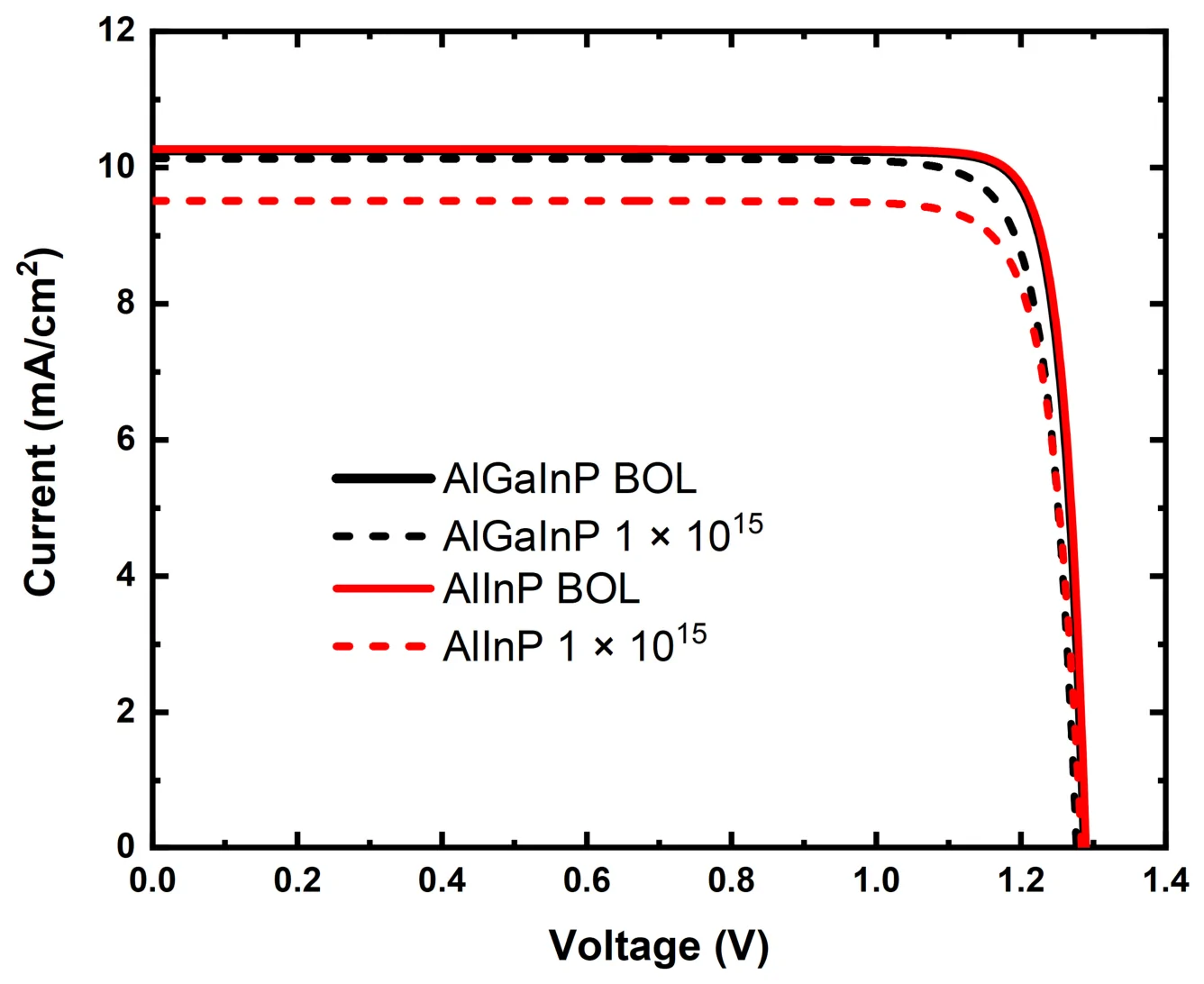
Simulated *J–V* characteristics of GaInP solar cells with different BSF layers before and after electron irradiation.

Figure 6 shows the simulated *J–V* curves of solar cells with the two types of BSF layers under electron irradiation. The simulated results reproduce well the degradation trends observed in the experiments, while the corresponding measured *J–V* data are shown in Figure 2. Irradiation introduces abundant deep-level defects and shortens the carrier lifetime within both device structures. Nevertheless, the AlGaInP BSF device maintains a favorable photogenerated carrier collection capability and exhibits only a mild *J_sc_* reduction. In contrast, the *J_sc_* of the AlInP BSF cell decreases monotonically with increasing irradiation fluence, and severe current attenuation occurs at a fluence of 1 × 10^15^ e/cm^2^, which is in good quantitative agreement with experimental measurements. This demonstrates that the AlInP BSF is more susceptible to electron irradiation-induced defects.

The overall degradation trend of the simulated *J–V* characteristics is consistent with our experimental observations, demonstrating that the established TCAD simulation framework can reproduce the experimentally observed relative degradation behaviors. Within this TCAD model, the total recombination losses originating from irradiation-induced defects are phenomenologically characterized by a degraded minority-carrier lifetime across distinct device regions. The minority-carrier lifetime degradation behavior of the GaInP absorber under 1 MeV electron irradiation in this TCAD simulation is consistent with reported simulation and experimental results [25]. The magnitude of lifetime reduction agrees reasonably with both previous TCAD outputs and the effective minority-carrier lifetime of GaInP top subcells [6]. In addition, DLTS-based defect investigations indicate that electron irradiation generates phosphorus vacancy–phosphorus interstitial Frenkel pairs and gallium-vacancy-related deep traps in GaInP, which serve as dominant SRH recombination centers and account for the shortening of the minority-carrier lifetime [36]. These reported defect characteristics support our trap-density-related lifetime degradation setup for the GaInP active layer. It should be noted that the consistency between simulation and experimental degradation behaviors corresponds to phenomenological calibration, rather than fully independent physical validation. This calibrated TCAD model is mainly adopted to interpret the underlying degradation mechanisms of the two BSF structures.

**Figure 7:**
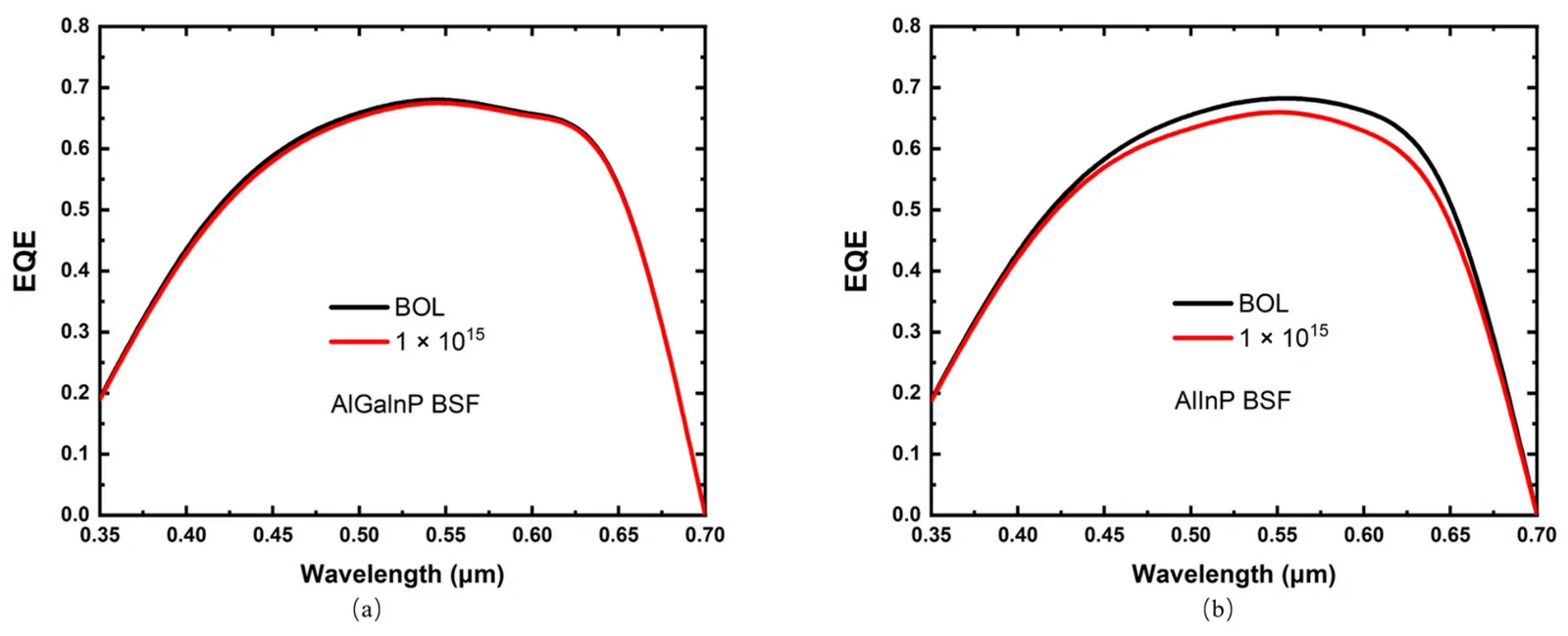
Simulated EQE spectra of GaInP solar cells with different BSF layers before and after electron irradiation: (**a**) AlGaInP BSF; (**b**) AlInP BSF.

Figure 7 displays the simulated EQE spectra. After high-dose irradiation, the EQE of the cell with an AlInP BSF degrades significantly by more than 12%, which mainly originates from severe carrier loss at the heterointerface together with enhanced SRH non-radiative recombination at irradiation-induced deep-level defects. In contrast, the long-wavelength EQE of the AlGaInP BSF cell barely decays, which is consistent with the better radiation-resistant properties of AlGaInP quaternary alloy. Due to numerical artifacts originating from the metal contact geometry and optical model simplifications elaborated in Section 2.3, the Sentaurus TCAD simulation yields lower *J_sc_* and EQE values compared with experimental measurements. This simulation focuses on the relative performance differences between the two BSF structures under electron irradiation. With identical modeling settings adopted for both structures, the observed comparative trends remain persuasive. The degradation trend of the simulated EQE spectra is fully consistent with that of the measured spectra, which further shows the rationality of the TCAD equivalent minority-carrier lifetime irradiation model from the perspective of spectral carrier collection.

It should be pointed out that our device-scale experiments and drift-diffusion TCAD simulations capture the macroscopic degradation behavior of BSF structures but cannot resolve atomic-scale defect generation parameters such as displacement threshold energies and defect migration barriers. To our knowledge, direct quantitative comparative data for these two parameters between AlInP and AlGaInP under 1 MeV electron irradiation are still absent in the published literature. Further atomic-scale calculations would be required to obtain these targeted atomic-scale parameters in future investigations.

## 4. Conclusions

In this work, 1 MeV electron irradiation experiments were performed on GaInP solar cells with AlGaInP and AlInP BSF layers. Through a combination of *J–V* and EQE characterizations, as well as Sentaurus TCAD simulations, the irradiation-induced degradation behavior and the underlying physical mechanisms of the two BSF structures were systematically explored. A distinct performance trade-off is identified between the two device configurations targeted for space photovoltaic applications. The AlGaInP BSF exhibits excellent radiation tolerance under high-fluence electron irradiation, albeit with a moderate band offset that limits its carrier confinement capability. In contrast, the AlInP BSF delivers a larger conduction-band offset and a higher initial V_oc_; however, it is vulnerable to irradiation-induced defect accumulation, resulting in severe performance degradation after electron irradiation. This study suggests that interfacial characteristics and the BSF structural configuration exert a considerable influence on the balance between static photovoltaic performance and the radiation endurance of GaInP solar cells. Future work will focus on interfacial passivation and composition-graded BSF optimization to simultaneously improve carrier confinement and radiation hardness. This work offers fundamental experimental evidence and theoretical guidance for the structural design of radiation-hardened GaInP top cells suitable for aerospace power systems.

## Figures and Tables

**Table 1:** Structural parameters of the two solar cell.

Layer	Composition	Type	Thickness (nm)	Doping Concentration (cm^−3^)
Substrate	GaAs	p	—	2 × 10^18^
Bottom contact layer	GaAs	p	300	2 × 10^18^
BSF-A	AlGaInP	p	30	2 × 10^18^
BSF-B	AlInP	p	30	2 × 10^18^
Base	GaInP	p	700	7 × 10^16^
Emitter	GaInP	n	80	4 × 10^18^
Window layer	AlInP	n	20	3 × 10^17^
Top contact layer	GaAs	n	300	5 × 10^18^

**Table 2:** Basic material parameters adopted in TCAD simulations.

Parameter	GaInP	AlGaInP	AlInP	GaAs
Bandgap energy (eV)	1.88	2.08	2.26	1.42
Relative permittivity	11.800	11.666	11.125	13.1
Electron effective mass	0.12584	0.12896	0.14144	0.067
Hole effective mass	0.521282	0.501297	0.446647	0.642

**Table 3:** Minority-carrier lifetime for AlGaInP-BSF and AlInP-BSF devices.

Fluence ($e/cm^{2}$)	AlGaInP-BSF τ (ns)	AlInP-BSF τ (ns)
0 (BOL)	50.00	50.00
5 × 10^13^	7.14	1.44
1 × 10^14^	3.85	0.73
5 × 10^14^	0.82	0.148
1 × 10^15^	0.41	0.074

## Data Availability

The original contributions presented in this study are included in the article. Further inquiries can be directed to the corresponding authors.
